# Supplementary material for: Transport Properties and Finite Size Effects in β-Ga2O3 Thin Films
Source: Sci Rep. 2019 Sep 11;9:13149. doi: 10.1038/s41598-019-49238-2 (PMC6739305; doi:10.1038/s41598-019-49238-2)
Supplement: Supplementary file 1 — Supplementary Information [file 41598_2019_49238_MOESM1_ESM.pdf]

Transport Properties and Finite Size Effects in β-Ga_2O_3 Thin Films

Robin Ahrling[1], Johannes Boy[1], Martin Handwerg[1], Olivio Chiatti[1], Rüdiger Mitdank[1], Günter Wagner[2], Zbigniew Galazka[2], and Saskia F. Fischer[1,*]

[1]Novel Materials Group, Humboldt-Universität zu Berlin, Newtonstraße 15, 12489 Berlin, Germany
[2]Leibniz Institute for Crystal Growth, Max-Born-Straße 2, 12489 Berlin, Germany
[*]sfischer@physik.hu-berlin.de

ABSTRACT

Thin films of the wide band gap semiconductor β-Ga_2O_3 have a high potential for applications in transparent electronics and high power devices. However, the role of interfaces remains to be explored. Here, we report on fundamental limits of transport properties in thin films. The conductivities, Hall densities and mobilities in thin homoepitaxially MOVPE grown (100)-orientated β-Ga_2O_3 films were measured as a function of temperature and film thickness. At room temperature, the electron mobilities ((115±10) $\frac{\mathrm{cm}^2}{\mathrm{Vs}}$) in thicker films (> 150 nm) are comparable to the best of bulk. However, the mobility is strongly reduced by more than two orders of magnitude with decreasing film thickness ((5.5±0.5) $\frac{\mathrm{cm}^2}{\mathrm{Vs}}$ for a 28 nm thin film). We find that the commonly applied classical Fuchs-Sondheimer model does not explain the contribution of electron scattering at the film surfaces sufficiently. Instead, by applying an electron wave model by Bergmann, a contribution to the mobility suppression due to the large de Broglie wavelength in β-Ga_2O_3 is proposed as a limiting quantum mechanical size effect.

Supplementary Information

S1 Scattering by Optical Phonons and Ionized Impurities

The equations to fit the different scattering mechanisms were used as follows (for more details see Oishi *et al.*[1]):
The scattering of electrons with optical phonons is described by

$$\mu_{\mathrm{OP}} = \frac{4\varepsilon_0 \pi\hbar^2 \left[\exp\frac{E_{\mathrm{OP}}}{k_{\mathrm{B}}T} - 1\right]\left(1 - 5\frac{k_{\mathrm{B}}T}{E_{\mathrm{g}}}\right)}{em^*\left(\frac{1}{\varepsilon_\infty} - \frac{1}{\varepsilon_{\mathrm{S}}}\right)\sqrt{2m^* E_{\mathrm{OP}}\left(1 + \frac{E_{\mathrm{OP}}}{E_{\mathrm{g}}}\right)}}, \tag{S1}$$

where E_{g} describes the gap energy, E_{OP} the energy of the optical phonons, ε_0 the vacuum dielectric constant, ε_{S} the low frequency dielectric constant and ε_∞ the high frequency dielectric constant.
The scattering of electrons with ionized impurities is described by

$$\mu_{\mathrm{II}} = \frac{128\sqrt{2}\varepsilon_{\mathrm{S}}^2 (k_{\mathrm{B}}T)^{3/2}}{\sqrt{m^*}Z^2 e^3 N_{\mathrm{II}}}\left(\ln(1+b) - \frac{b}{1+b}\right)^{-1}, \tag{S2}$$

with N_{II} as density of ionized impurities, Z as the electrical charge of the scattering centers and b being

$$b = \frac{96\pi^2 \varepsilon_{\mathrm{S}}\varepsilon_0 m^*}{N_{\mathrm{II}}}\left(\frac{k_{\mathrm{B}}T}{\hbar e}\right)^2 \tag{S3}$$

S2 Fit parameters

The least-square-fits of the mobility data under consideration of electron scattering with phonons and ionized impurities yielded the following parameters, see table S1.

Table S1. Least-square-fit parameters of the mobility fits considering electron scattering with phonons and ionized impurities. Values for the 60 nm 40 nm and 28 nm sample are not given,since the scattering is not dominated by electron phonon interaction, but instead the finite size effectsplay a leading role.

sample thickness	effective mass m^*	phonon energy E_{OP} [meV]	dielectric constant ε_S
bulk	0.24 ± 0.03	39 ± 5	14 ± 2
225 nm	0.26 ± 0.03	38 ± 5	11 ± 2
205 nm	0.22 ± 0.03	30 ± 5	14 ± 2
160 nm	0.28 ± 0.03	58 ± 5	13 ± 2
155 nm	0.29 ± 0.03	50 ± 5	10 ± 2

S3 Application of the Fuchs-Sondheimer Model

The commonly used model to explain a reduction in mobility in thin metal films as a function of film thickness is the Fuchs-Sondheimer model. For the case of $l \ll t$ it can be approximated to[2,3]

$$\frac{\mu_f}{\mu_{bulk}} = \frac{1}{1+\frac{3l}{8t}(1-p)}, \tag{S4}$$

describing the ratio of the mobility in thin films μ_f and in bulk μ_{bulk} in dependence of the ratio of the mean free path l and the film thickness t. p denotes the specularity parameter, ranging from $p = 0$ for completely diffusive and $p = 1$ for completely specular scattering. The maximum mobility reduction takes place when scattering is completely diffusive.
As can be seen in figure S1, a fit with eq. S4 was carried out. Even for the most extreme assumptions, a specularity parameter of $p = 0$ and a constant mean free path of bulk of $l_{const} = 6$ nm, the Fuchs-Sondheimer model can not explain the mobility reduction observed in the homoepitaxial β-Ga_2O_3 thin films.

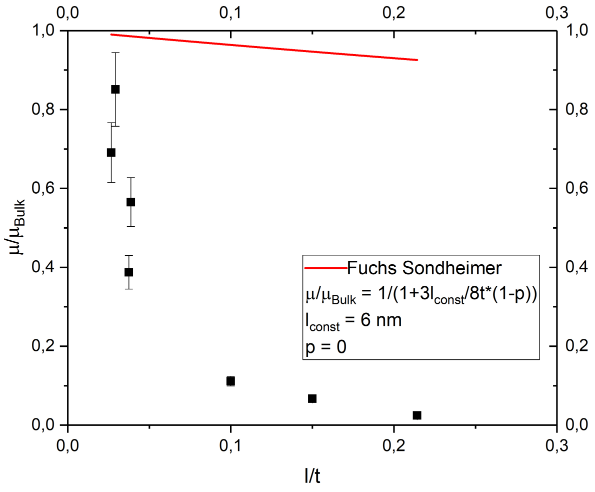

Figure S1. Thin film mobility relative to bulk mobility vs. ratio of mean free path to film thickness. The red line shows a fit after the Fuchs-Sondheimer model for a constant value of $l = 6$ nm. The observed mobility reduction is much larger than the model could explain.

S4 Application of the Bergmann Model

In contrast to the Fuchs-Sondheimer model, the Bergmann model[4] also takes into account the wave properties of electrons. It was developed for samples with an infinite intrinsic mean free path and describes a different dependence for the mobility reduction with decreasing film thickness. Here, the wavelength of the electrons plays an important role. In contrast to the intrinsic mean free path l_{int}, the effective mean free path l in the Bergmann model is not infinite but is reduced as the mobility gets reduced. Since the electron wavelength becomes comparable to the film thickness in the thin semiconducting samples examined in this work, the wave nature of the electrons becomes important and the Bergmann model can be considered for the explanation of the mobility suppression.

References

1. Oishi, T., Koga, Y., Harada, K. & Kasu, M. High-mobility β-Ga_2O_3 ($\bar{2}01$) single crystals grown by edge-defined film-fed growth method and their Schottky barrier diodes with Ni contact. *Appl. Phys. Express* **8**, 031101 (2015).
2. Sondheimer, E. The mean free path of electrons in metals. *Adv. Phys.* **1**, 1–42 (1952).
3. Sondheimer, E. The mean free path of electrons in metals. *Adv. Phys.* **50**, 499–537 (2001).
4. Bergmann, G. Conductance of a perfect thin film with diffuse surface scattering. *Phys. Rev. Lett.* **94**, 106801 (2005).
